# Long-term results of staged management of complex lisfranc and chopart injury: a retrospective cohort study and systematic literature review

**DOI:** 10.1007/s00068-024-02747-w

**Published:** 2025-01-24

**Authors:** Esmee W. M. Engelmann, Jens A. Halm, Tim Schepers

**Affiliations:** 1 Department of Trauma Surgery, Amsterdam University Medical Centers, Location AMC, Meibergdreef 9, Amsterdam, 1105 AZ The Netherlands; 2 Amsterdam Movement Sciences, Amsterdam University Medical Centers, Meibergdreef 9, Amsterdam, 1105 AZ The Netherlands

**Keywords:** Lisfranc, Chopart, Midfoot, Tarsometatarsal injuries, Foot trauma, Complex midfoot trauma

## Abstract

**Purpose:**

The aim was to assess the long-term functional outcome and quality of life after staged surgical treatment of complex Lisfranc and Chopart injuries in a patient cohort, and to perform a systematic review of the literature.

**Methods:**

A retrospective cohort of all trauma patients with complex Lisfranc and/or Chopart injuries treated at our level 1 trauma center between July 1, 2010, and July 1, 2020 with *≥* 3 years follow-up was analyzed in terms of management, complications, and patient-reported outcomes (American Orthopaedic Foot & Ankle Society midfoot score, AOFAS and Foot Function Index, FFI). A systematic review of the literature (according to PRISMA 2020 guidelines) was performed of studies published between January 2000 to April 2024. Inclusion criteria were acute, complex Lisfranc and/or Chopart injury, staged treatment, age *≥* 17 years, patient-reported outcome measures and *≥* 1 year follow-up.

**Results:**

Fifteen patients with a median follow-up of 6.6 years (interquartile range 4) were included. First stage treatment involved temporary K-wire fixation (*n* = 11), debridement (open fractures, *n* = 4), external fixation (*n* = 4) and decompression fasciotomy (*n* = 3). Second stage included primary arthrodesis (*n* = 4), open reduction and internal fixation (*n* = 7) or external fixation (*n* = 1). The median number of surgeries was 3 (2–13). Infection was seen in 5/15 patients; 7/11 patients underwent secondary arthrodesis and one patient underwent amputation due to chronic pain. Overall AOFAS was fair (54.5) and five patients had poor outcome (AOFAS < 49). Three full-length articles were included in the systematic review, reporting fair to good outcome. Risk of bias was serious and certainty of evidence very low.

**Conclusion:**

Surgical management of complex Lisfranc and Chopart injuries is challenging, functional outcome was poor to fair, postoperative complication rates are high, and secondary salvage arthrodesis was required in two thirds of ORIF patients.

**Level of evidence:**

IV

**Supplementary Information:**

The online version contains supplementary material available at 10.1007/s00068-024-02747-w.

## Introduction

Among midfoot injuries, combined Lisfranc and Chopart fracture-dislocations may be the most challenging injuries to treat. The Chopart joint consists of the talonavicular and calcaneocuboid joints. Distally, the Lisfranc joint is defined as a tarsometatarsal joint complex, including all cuneiforms, the cuboid and articulations with the metatarsal bases. Together, these constitute the osseous and ligamentous foundation of the longitudinal and transverse foot arches [1].

The severity of Lisfranc and Chopart injuries varies from subtle to complex, depending on the extent of damage to bones, joints and soft tissues affected. In high energy Lisfranc injuries, the majority of patients has concomitant foot fractures including Chopart injuries (84%), displaced intra-articular fractures (59%) and involvement of all five rays (23%) [2]. The relation between the midfoot articulations, and thus the effect of combined Lisfranc and Chopart injuries on foot biomechanics, is best understood by use of the column theory [3]. The medial column includes the navicular, medial cuneiform and first metatarsal. The middle column consists of the second and third metatarsal and cuneiforms. The lateral column includes the calcaneus, cuboid, fourth and fifth metatarsals.

In general, anatomical reduction is the most important predictor of good results after Lisfranc and Chopart injuries [4–6]. In clinical practice, the appropriate treatment depends on the degree of dislocation, the number of affected joints, soft tissue injury, articular cartilage damage and ligamentous injury [1]. Especially in patients who suffered high-energy trauma, other (soft tissue) injuries may influence timing and outcome [2, 7, 8].

An adequate initial approach may provide the optimal result as compared to secondary salvage procedures [9]. However, this can be highly challenging in case of severe Lisfranc and Chopart injuries with associated soft tissue problems such as open fractures or compartment syndrome. The goal of the initial surgery in such cases is to reduce major dislocations and, wherever possible, improve local soft tissue conditions to later perform additional surgery aimed at the best anatomical reduction achievable [5, 10]. These patients usually require multiple surgeries including temporary reduction and fixation, decompression fasciotomy, (staged) debridement, and (flap) coverage. Multidisciplinary management in a strategical sequence is required for foot function rescue.

For both patient and surgeon, it is important to be informed about the rate of complications and the potential functional outcome in case of complex Lisfranc and Chopart injuries. Interestingly, for high-energy injuries involving joints at other locations, such as pilon or tibial plateau fractures, it has been demonstrated that staged management can significantly improve patient outcome [11–14]. At current, there is no literature regarding long-term patient-reported outcome of staged management of complex Lisfranc and Chopart injuries. The aim of this study was therefore to perform a systematic literature review to provide an overview of the state of evidence, and to assess the long-term functional outcome and quality of life after staged surgical treatment in a consecutive series of patients.

## Materials and methods

### Cohort study design and patients

A definition of complex Lisfranc and Chopart injury was not found in literature. In this study, complex Lisfranc and Chopart injury were defined as the most severe type of injury, often after high energy trauma, characterized by a combination of bony, soft tissue and/or neurovascular injury and associated with articular involvement, fracture comminution and injury beyond the extent of one joint (therefore also referred to as complex midfoot injury) [15, 16]. It imposes a high risk in terms of morbidity due to complications and limited functional outcome. A retrospective cohort of trauma patients with complex Lisfranc and Chopart injuries who were treated in staged manner at our level 1 trauma center between July 1, 2010, and July 1, 2020 was analyzed. Institutional review board approval (W19_247) and written informed consent were obtained.

Inclusion criteria were: complex Lisfranc and Chopart injury, acute injury, staged treatment, age at trauma above 17 years and at least three years of follow-up. Patients who underwent staged treatment (temporary Kirschner wires, wound debridement, ORIF in multiple stages, external fixation, decompression fasciotomy) were included. Exclusion criteria were patients with missed or longstanding Lisfranc joint injury and patients with a history of foot ankle surgery. Patient-related, clinical and radiographic data were extracted from the electronic hospital database. Included patients were evaluated at the outpatient clinic and received a questionnaire including AOFAS and FFI amongst others.

### Variables

Variables included were age at injury, gender, BMI, mechanism of trauma, injury pattern, number of injured rays and joints, Myerson, Column Involvement Severity System (CISS), Zwipp and Gustilo classifications, and concomitant ipsi- or contralateral lower extremity injuries [17–20]. Data was collected on the type of treatment, follow-up, complications, functional outcome and quality of life. Complications were defined as fracture-related infections (FRI) and delayed (> 6 months), mal- (healing in abnormal position) or non-union (> 9 months without signs of healing for 3 months). Functional outcome was assessed using the Foot Function Index (FFI, best score 0 points), and the American Orthopaedic Foot and Ankle Society midfoot score (AOFAS, best score 100 points). The AOFAS score was divided into groups according to the literature: a score of 90–100 was graded as an excellent result; 75–89 as good; 50–74 as fair, and less than 49 points was graded as a failure or poor outcome. Assessment of perceived general health was done using a Visual Analogue Scale (VAS) of 0 to 100, in which 100 represented excellent general health (EQ-VAS). Patient satisfaction was also measured using the VAS of zero to 10, in which 10 represents the best possible satisfaction.

### Statistical analysis

The statistical analysis was performed using the Statistical Package for the Social Sciences (SPSS) version 29 (SPSS, Chicago, IL). Numeric data are expressed with means with standard deviation or median with interquartile range (after ruling out normal distribution). Categorical data are shown as numbers with percentages. Independent sample two-tailed t-test with a significance level of 0.05 were used to compare means.

## Literature review

**Fig. 1:**
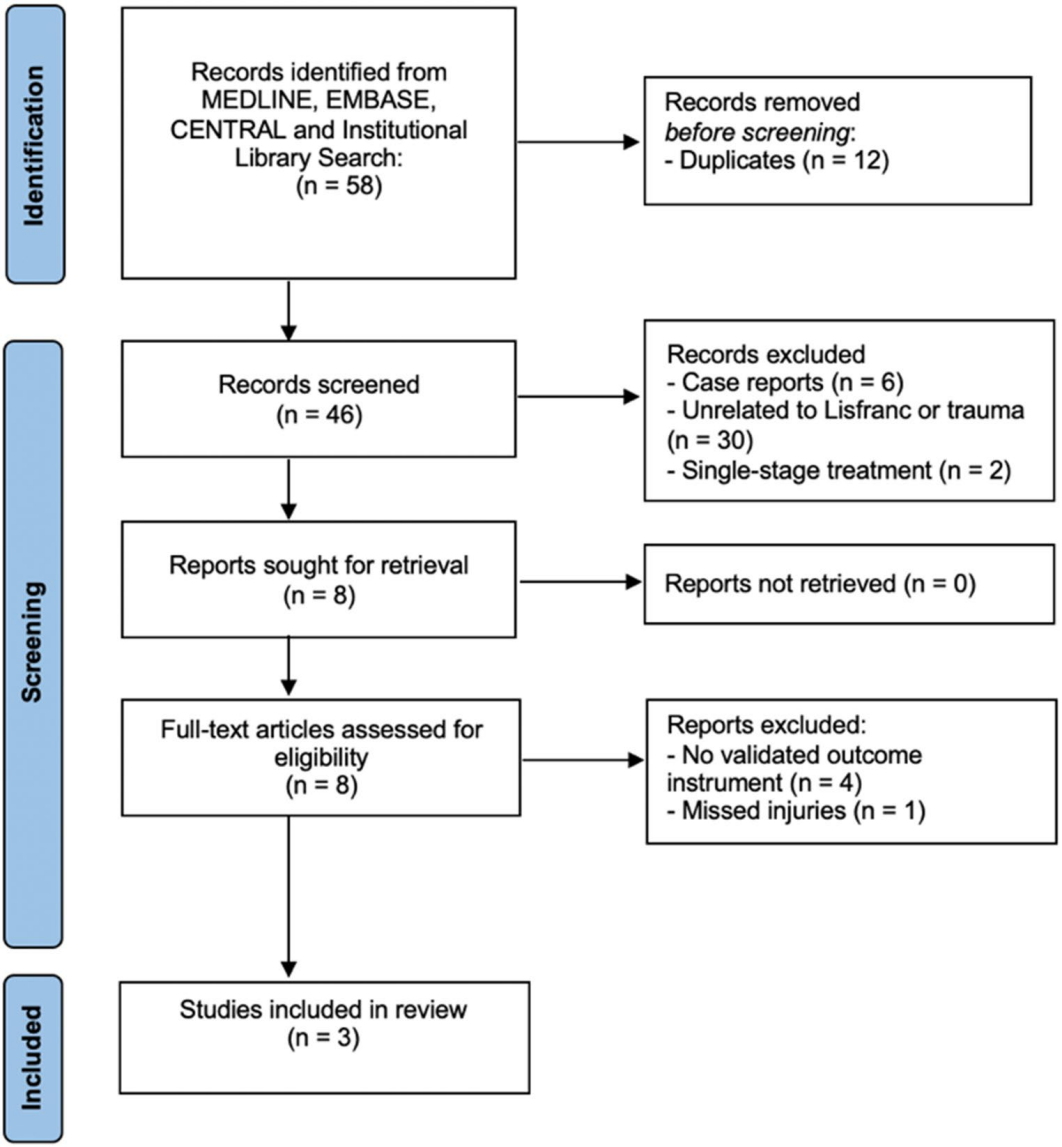
PRISMA flow chart

A systematic review of the literature, in adherence to the PRISMA guidelines [21], was performed of the following databases using the OVID search engine: MEDLINE, EMBASE and CENTRAL databases (January 2000 to April 2024) using the terms “staged [tiab]” and “Lisfranc [tiab]” or “Chopart” [tiab] or “midfoot [tiab]”. All sources were last consulted on November 25th, 2024. Because of the advances in therapeutic strategies of complex midfoot injuries, studies prior to 2000 were excluded. Two authors (EWME and TS) performed the systematic review and consequent data extraction independently. Results from all databases were combined and duplicates removed. In case of disagreement, a third independent reviewer was consulted (JAH) to provide consensus on inclusion. Criteria for the selection of articles are outlined in Table 1. The search strategy and selection process are illustrated in Fig. 1. Risk of bias was assessed using the ROBINS-I V2 tool and certainty of evidence based on GRADE, according to the same principle (EWME and TS independently, complete consensus) [22, 23].

**Table 1:** Selection criteria

	Inclusion	Exclusion
1	Studies involving staged management of Lisfranc and/or Chopart injuries	Studies that included < 10 patients
2	Acute injuries (interval from trauma to treatment < 6 weeks)	Inability to isolate complication and functional outcome results
3	Patients must be adults (age > 17 years)	Mean follow-up < 12 months
4	Validated functional outcome scores (AOFAS, FFI, VAS, MFA, Weber)	Non-English-language studies
5	Studies published between 2000 and 2024	Full article not provided

The study by Richter et al. [4], may appear to meet inclusion criteria, however treatment was not staged and the paper was therefore excluded.

### Variables

Data on the year of publication, type of study, number of patients, gender, age, mechanism of injury, type of fracture (anatomy), treatment, postoperative protocol, primary outcome, complications as reported by authors, functional outcome and follow-up period were extracted from the reports by EWME, collected in an Excel data sheet, and checked by TS and JAH independently. Only studies that applied validated functional outcome instruments, such as the Foot Function Index (FFI), American Orthopaedic Foot & Ankle Society midfoot score (AOFAS), Weber functional outcome score or Musculoskeletal Function Assessment (MFA) were included. No additional data had to be obtained from study investigators. For each outcome, the effect measure presented in this review was the measure reported by the authors. This is a narrative systematic review as data conversions or synthesis were not considered feasible due to high heterogeneity and serious risk of bias (Appendix I).

## Results

### Patient and injury characteristics

Out of 244 screened patients with Lisfranc and Chopart injuries treated in our hospital between July 1, 2010, and July 1, 2020, 15 patients with complex Lisfranc and Chopart injuries who underwent staged management were identified (6.1%). Patient and injury characteristics are reported in Table 2. Median age was 34.5 years (IQR 16). All patients were diagnosed using both conventional radiography and CT imaging. Two patients met the Berlin definition of polytrauma (injuries with an Abbreviated Injury Scale score of *≥* 3 in *≥* 2 body parts combined with the presence of *≥* 1 physiological risk factors including age, Glasgow Coma Scale, hypotension, acidosis and coagulopathy) [24].

**Table 2:** Patient and injury characteristics

Pt	Gender	Age	MoI	Ipsilat. fractures	Open	Myerson	CISS	Rays	Zwipp	Contralat. fr.
1	M	29	MVC	TMT 1 destruction (crush), MT 2–4, cun 1–3, cuboid, calcaneus	No	C2	M3C1L2	TMT 1–4	Type 6 (3,5)	Trimalleolar ankle
2	M	40	Fall (height)	MT 1–5, calcaneus	Gr 3a	B2	M2C3L3	TMT 1–5	NA	Calcaneus
3	M	43	Fall (height)	MT 1–5	Gr 3a	A	M1C2L3	TMT 1–5	NA	Medial pilon
4	M	37	MVC	MT 1–4, cun 2–3, cuboid	No	B2	M1C3L3	TMT 1–4	NA	No
5	M	35	Blast	MT 1–2, cun 1–2, cuboid, phalanx 2–3	Gr 3a	A	M3C3L1	TMT 1–5	NA	No
6	F	31	Fall (LET)	Navicular, cuboid, cun 1–3, MT 2–5	No	A	M1C3L3	TMT 2–5	Type 6 (4,5)	No
7	F	49	Crush	MT 1–5	No	C2	M3C3L1	TMT 1–5	NA	No
8	F	80	Fall (LET)	MT 2–3, cun 1–3, cuboid	No	A	M1C3L1	TMT 1–5	NA	No
9	M	31	Crush	MT 1–2, cun 1–3, cuboid	No	B2	M1C2L0	TMT 1–2	NA	No
10	F	65	Fall (LET)	MT 2–5	No	A	M1C3L3	TMT 2–5	NA	No
11	M	18	Crush	MT 1–3, cun 1–2, navicular, calcaneus	No	B2	M2C2L0	TMT 1–3	Type 6 (1,3,4)	No
12	M	69	Fall (height)	MT 4–5, cun 1–3, navicular, cuboid	No	B2	M0C0L2	TMT 4–5	Type 6 (1,4,5)	No
13	M	34	Fall (height)	MT 4, navicular, cuboid, calcaneus, talus	No	NA	NA	NA	Type 6 (2–5)	No
14	M	27	Crush	MT 2–5, cun 1–3, navicular, calcaneus, cuboid, distal fibula	Gr 3a	B2	M1C3L3	TMT 2–5	Type 6 (3–5)	No
15	M	34	MVC	MT 3–5, navicular, cuboid, calcaneus, talus, bimalleolar ankle	No	NA	M0C1L1	NA	Type 6 (2–5)	No

Age at trauma in years. Abbreviations: MoI, mechanism of injury; MVC, motor vehicle collision; LET, low energy trauma; MT, metatarsal; cun, cuneiform; NA, not applicable

### Management

In most patients, initial (first stage) treatment involved temporary, non-threaded K-wire fixation (*n* = 11). In case of open fractures, debridement and wash-out followed by wound closure was performed prior to K-wire placement (*n* = 4). Four patients were treated by temporary external fixation and three patients required decompression fasciotomy due to compartment syndrome. Median interval between first and second stage surgery was 6 days (IQR 17). There were no strict criteria for timing of the second stage, this was mostly dependent on general patient physiology, soft tissue conditions (such as swelling, infection) and other injuries.

**Fig. 2:**
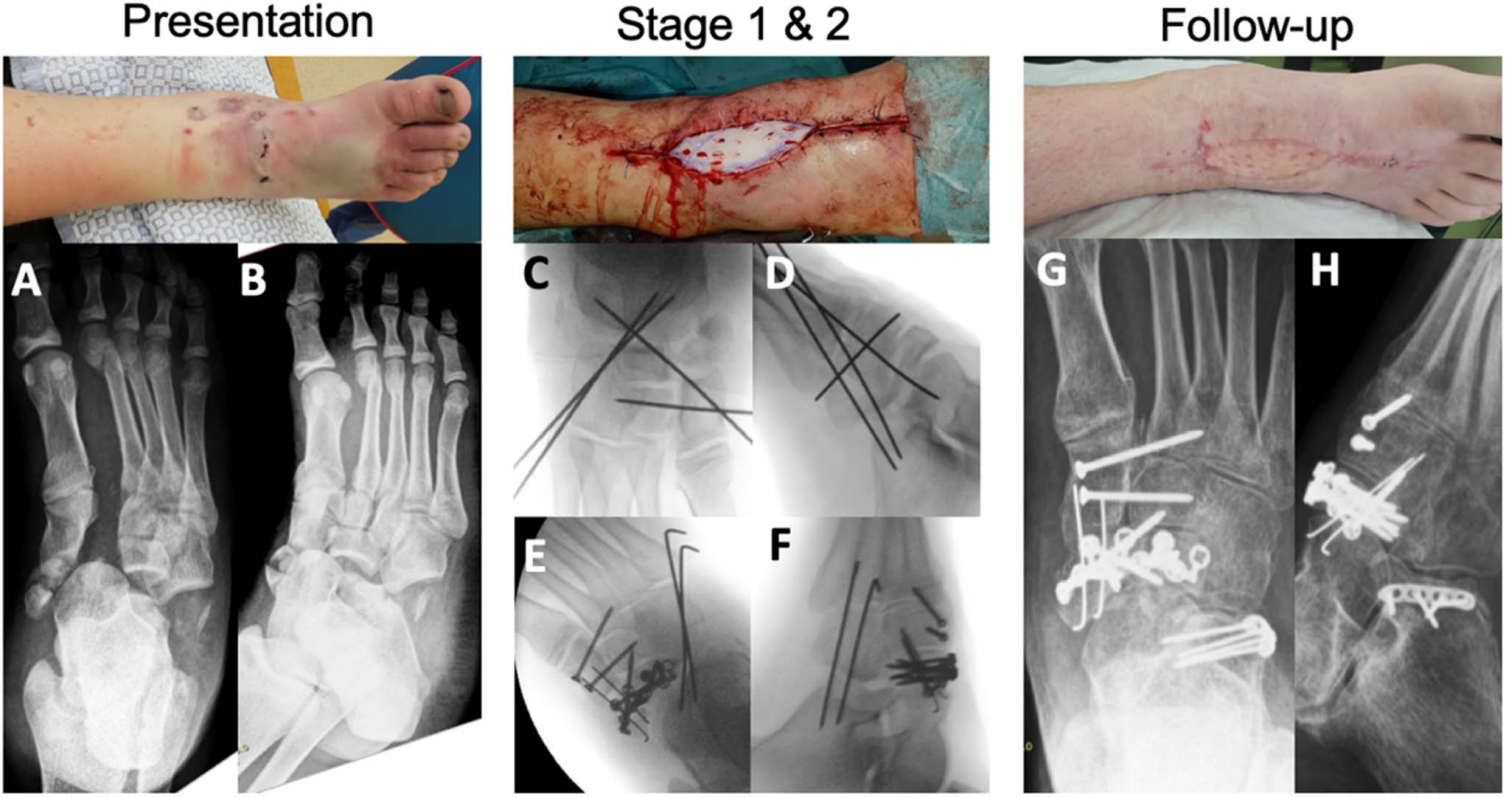
18 years old patient with combined Chopart and Lisfranc injury pre- and postoperatively

**Fig. 3:**
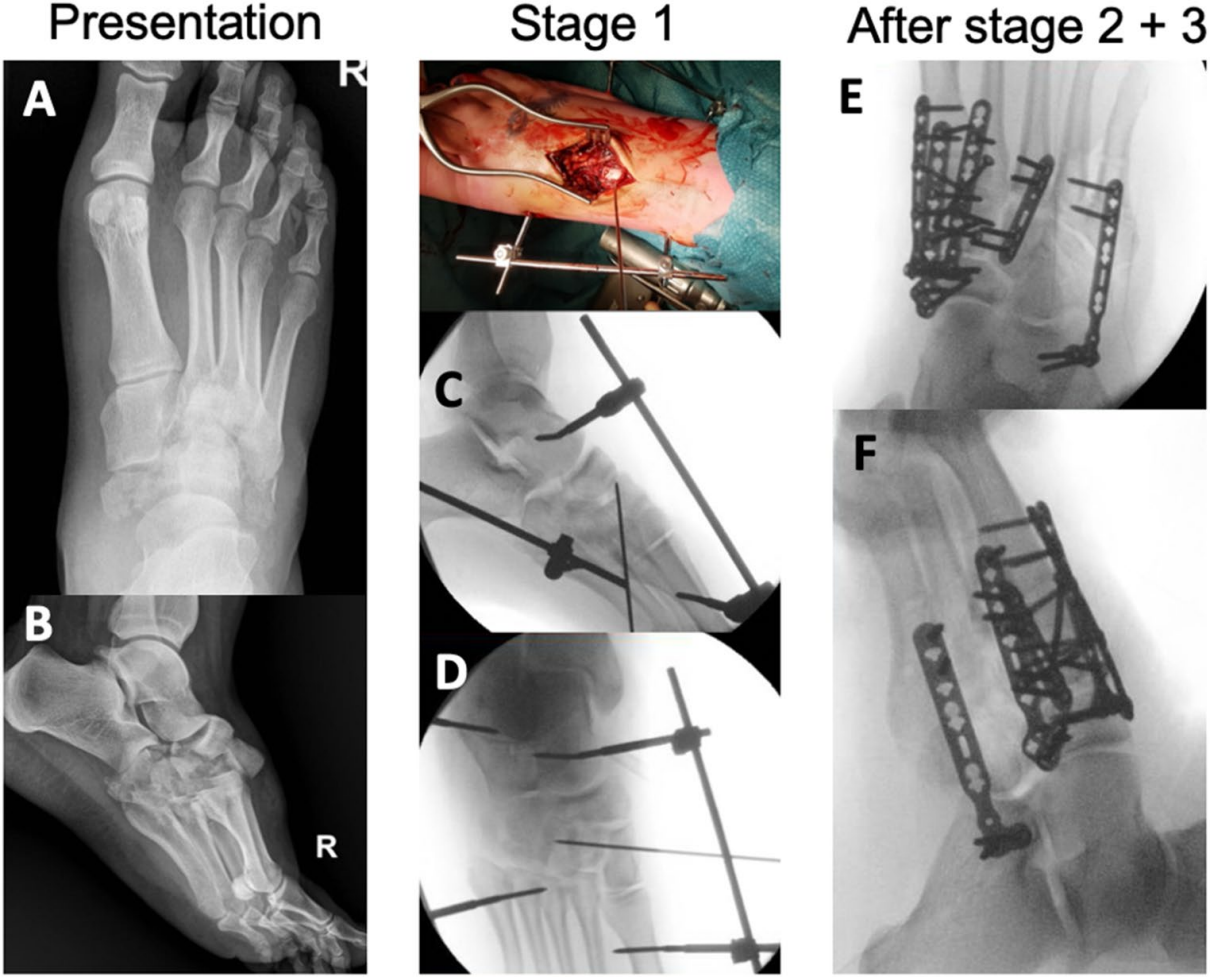
31 years old female patient with combined Chopart and Lisfranc injury pre- and postoperatively

The second stage surgical plan was tailor-made. In general, it was aimed at open reduction and joint sparing, if necessary with bridge plating. Candidates for PA were patients with non-reconstructable joints, mostly due to severe crush injury or comminution (> 50% of articular surface). During second stage, four patients were treated with primary arthrodesis (PA), open reduction and internal fixation (ORIF, *n* = 7) or external fixation (*n* = 1). Percutaneous K-wires were left in situ for six weeks in three patients. With regard to soft tissue coverage, three patients (ORIF) required a split skin graft, two patients (PA and ORIF) received a full thickness graft, one patient (K-wires) required a free flap (anterolateral thigh) and one patient was treated with negative pressure wound therapy (NPWT). The general postoperative rehabilitation protocol was 6 to 8 weeks of non-weightbearing in a cast, but also dependent on the presence of K-wires or external fixator and the condition of soft tissues (including graft/flap). Overall, the median number of surgeries was 3, spread as; 2 (*n* = 4), 3 (*n* = 4), 4 (*n* = 2), 5 (*n* = 2), 6 (*n* = 2), 13 (*n* = 1, crush injury, including 7 surgeries for NPWT). Two cases are presented in Figs. 2 and 3.

Case of an 18-year old male who sustained crush injury of the foot due to the wheel of a heavy truck (Table 2, pt 11). This is a complex midfoot injury with multiple fracture-dislocations in the Chopart and Lisfranc joints (A, B). At presentation, there was severe swelling and hematoma with superficial lacerations, no neurovascular injuries. Stage 1 (2 h after presentation) consisted of decompression fasciotomy and temporary K-wire fixation of the Chopart and Lisfranc joints (C, D). Stage 2 (4 days later) was ORIF of the medial column (Lisfranc screws) and navicular fracture (E, F). Wound closure was achieved by abdominal full thickness graft. Stage 3 (2 weeks later) was ORIF of the calcaneocuboid joint (G, H). Fracture union was achieved without complications. At 5 years follow-up, the patient returned to work as a truck driver without pain and with good foot function (AOFAS 80, FFI 9).

Case of a 31-year old female who fell from a bicycle (Table 2, pt 6) and suffered complex Lisfranc injury (A, B). Stage 1 (directly after referral, 3 days after trauma) was open reduction, K-wire fixation of the intermediate cuneiform and external fixation (C, D). Stage 2 (9 days later) was ORIF of the medial column: TMT 1 double bridge plating due to injury severity, TMT 2 fusion, TMT 3 plate. This was followed by stage 3 (4 days later) which consisted of ORIF of the lateral column using a bridge plate from the calcaneus to TMT 4 and 5, spanning the comminuted cuboid bone (E, F). The medial and lateral bridge plates were removed 6 months later.

### Complications

Fracture union was achieved in 14 out of 15 patients (*n* = 1 nonunion of primary TNJ arthrodesis due to deep infection). Fracture-related infection was seen in 33.3% (*n* = 2 superficial after first stage K-wire fixation, *n* = 1 deep after first stage K-wire fixation, *n* = 1 deep after second stage ORIF, *n* = 1 chronic osteomyelitis after crush injury). All were treated with oral (*n* = 2 superficial) or intravenous antibiotics (*n* = 3) and surgical debridement. Implants were removed in six patients (one related to infection). Seven out of eleven patients (63.6%) not treated with PA underwent secondary arthrodesis (SA) due to painful osteoarthritis at a median interval of 18 months (IQR 14) after initial surgery. Due to chronic pain, severe functional limitation and related morbidity, one patient eventually underwent a below knee amputation 6 years after trauma.

### Functional outcome and patient perceived health

Questionnaires were sent to all patients, of whom 100% responded. Functional outcome and patient-reported outcome measures per group are demonstrated in Table 3. Overall median AOFAS was only fair (54.5, IQR 29.5). Return to (modified) work rate was 50% (*n* = 7/14 patients who worked prior to trauma). Four out of eleven patients (36.4%) were able to return to sports. With the numbers available, no significant difference could be detected in functional outcome, treatment satisfaction and perceived health between subgroups.

**Table 3:** Functional outcome and patient-reported outcome measures

	Staged management (*n* = 15)
Follow-up time (years)	6.6 (4)
AOFAS	54.5 (29.5); fair good (*n* = 3) fair (*n* = 7) poor (*n* = 5)
FFI	43.3 (38.9)
Perceived health	7.3 (1.8)
Treatment satisfaction	8 (1.5)

Results in median (IQR). Abbreviations: AOFAS, American Orthopaedic Foot & Ankle Society Score; FFI, Foot Function Index

### Literature review

Three full-length articles were included (Fig. 1). All studies were retrospective cohort studies from China [24–26]. Detailed information of selected studies is displayed in Table 4.

**Table 4:** Summary of findings

Author	Year	Type	*N*	Gender	Age (y)	Details	Functional outcome	Complications	Follow-up (m)	Risk of bias	GRADE
Gu [24]	2017	RS	18	13 male, 5 female	42.2 (21–67)	- Gustilo-Anderson grade 3 open Lisfranc injuries, two-stage surgery (*n* = 18) − 1st stage: debridement and temporary K-wire fixation < 8 h. VAC for 7 days in pt with soft tissue defect. Antibiotics 3 days. − 2nd stage: K-wires for 6 weeks or removed and replaced with 4.3 mm cannulated screws or minifragment plate. Mean interval 9 (5–15) days, (local) flap or skin graft (*n* = 4)	- Median AOFAS: 74.4 *±* 8.7 - Median VAS: 2.2 *±* 1.8 − 64.3% return to work (*n* = 9/14)	− 14.3% SA (*n* = 2) - No nonunion, malunion, infection	Median 48 (36–60) 22.2% lost (*n* = 4)	Serious	Very low
Liu [25]	2020	RS	21	16 male, 5 female	Mean 44.4 (24–69)	- Open Lisfranc fracture-dislocations - 28.6% additional Chopart injury (*n* = 6) - Two-stage surgery: 1st stage bilateral spanning external fixator, VAC if required. - 2nd stage: removal external fixator, then ORIF (*n* = 14) or PA (*n* = 7). Mean interval 18.6 days (8–48)	- Mean AOFAS: 76.3 (63–97) - Average VAS: 2.4 (0–5)	− 42.9% traumatic arthritis after ORIF (*n* = 6/14) − 4.8% deep infection (*n* = 1), 1st stage − 9.5% superficial infection (*n* = 2), 1st stage	Average 15.4 (12–24)	Serious	Very low
He [26]	2022	RS	48	23 male, 25 female	Mean 49.5	- Closed Lisfranc injuries - Group A, *n* = 23: staged surgery, emergency reduction at 4–8 h after injury, second stage after 6–10 days, ORIF and TMT 1 fusion - Group B, *n* = 25: elective ORIF after swelling subsided, 10–20 days after injury	- Mean AOFAS: 86.87 *±* 4.24 (A) vs. 71.72 *±* 5.46 (B) - Mean VAS: 1.91 *±* 0.78 (A) vs. 3.20 *±* 1.17 (B)	- Traumatic arthritis (*n* = 2, group B) - No SA - Infection not described	Mean 18 (12–24)	Serious	Very low

Abbreviations: RS, retrospective study; y, years; m, months; VAC, vacuum assisted closure

## Discussion

Due to the severity of complex Lisfranc and Chopart injuries, surgical management is challenging, postoperative complication rates are high, average functional outcome was only fair, and secondary salvage arthrodesis was performed in the majority of patients after second-stage ORIF. Although staged surgical approach has been widely used in lower extremity injuries, only three previous studies with patient-reported outcomes after staged Lisfranc management and no studies on staged Chopart management were found in the literature. Risk of bias was serious and the certainty of the body of evidence very low. In fact, our cohort represents the first European study on this topic. Gu et al. also reported fair outcome, whereas Liu et al. reported good outcome [25, 26]. He et al. found good outcome in the group managed with staged treatment, compared to fair outcome in the patients that underwent one-stage elective ORIF [27]. However, based on the patient characteristics described, the aforementioned studies were not comparable to our cohort with the exception of age at presentation. Firstly, patients in the literature review underwent either one- or two-stage management whereas the median number of surgeries in our cohort was 3. Secondly, there was a variation in the soft tissue status (closed and open fractures), the injury or specific fracture patterns were not mentioned but high heterogeneity in terms of fracture-dislocation patterns and column involvement seems likely. Comparing the outcome scores of these three studies was not done in further detail because of heterogeneity in patient characteristics, injury severity and extent of the (concomitant) injuries. In addition, timing of surgery largely depends on soft tissue conditions, such as swelling, contamination, infection or other damage. Consequently, it is challenging to compare different treatment protocols. The best surgical strategy in terms of when and how to treat complex Lisfranc and Chopart injuries thus remains a topic of debate [1, 7, 8].

In general, soft tissue swelling is minimal during the first 6 to 8 h after injury (first window of opportunity) and between 7 and 14 days after injury (second window of opportunity) [27]. As seen in literature and based on general principles of (intra-articular) fracture reduction, it may be advisable to perform the first-stage of treatment including debridement, preliminary reduction and temporary fixation during the first window. Hereafter, Lisfranc and Chopart injuries with severe dislocation may develop increasing swelling, resulting in blisters or compartment syndrome (due to rupture of the anastomosis of plantar circulation and the intermetatarsal branch of anterior tibialis artery) [28]. As seen in literature and described in our cohort, early soft tissue coverage and restoration of column alignment are the main components of initial management. Temporary fixation can be achieved by either external fixator or Kirschner wires outside of the zones of injury and consequent definitive surgery. Kadow et al. described the use of external fixators to restore column length and alignment in the medial and lateral column [10]. It allows a more rigid and stable construct than Kirschner wires and if needed may be used as a form of definitive treatment [7, 29, 30]. Biomechanical proof of this concept is lacking in injuries with dislocation of all five rays. Alternatively, the use of Kirschner wires was suggested by Herscovici et al. as most applicable for the stabilization of the central column [5]. Infection of pin tracks is the main complication of both methods. Negative pressure wound therapy (NPWT) is effective as temporary coverage of soft tissue defects between surgeries, for accelerating local granulation tissue for primary healing or as wound bed preparation, and in treatment of local infection by removing fluids [6, 31–33]. Definitive coverage may be achieved by grafts (split skin, full thickness) or flaps.

In the second phase of treatment, the options are ORIF (joint preservation) or primary arthrodesis. If the patient or wound does not become amenable to surgery (because of other injuries or soft tissue problems), the temporary fixation may be used as definitive treatment. Since emergency ORIF is prone to postoperative wound complications, it is rarely used in clinical practice [27]. As an alternative to multiple surgeries, Qu et al. described a single-stage protocol in 20 patients with severe open Lisfranc injuries [34]. They were treated with wound debridement, internal fixation with K-wires (mean number 7.4, range 5–13) and NPWT during one-stage surgery. K-wires were removed after 6–8 weeks (lateral column) or 3–4 months (medial and central columns). Median AOFAS at 12 months was 78.2 (range 68–95). Feng et al. reported a median AOFAS of 75.8 (range 43–98) in a series of 15 patients with missed Lisfranc and Chopart injuries, treated with staged reduction (first stage external fixator) [35]. If ORIF is performed within 6 weeks of Lisfranc injury, outcome scores of patients are similar to those who underwent acute surgery [36]. Significantly worse outcomes have been reported when surgery was delayed more than 6 weeks [17, 37]. This may be related to poor soft tissue condition (scarring, contraction), articular cartilage damage caused by joint malalignment or osteoporosis [38, 39].

Interestingly, previous studies demonstrated that ORIF of the TMT joint does not always result in a true anatomic correction, leading to painful osteoarthritis (rates reported up to 94%) that may require secondary arthrodesis (SA) [39, 40]. There is an increasing body of evidence that PA may be the better option in selected patients because of reduction in secondary surgery rate, implant removal, chronic pain and higher functional outcome [41–44]. In a recent review comparing PA versus ORIF in patients with high-energy Lisfranc injuries, Godoy-Santos et al. found that PA showed better functional scores [45]. No statistically significant difference in terms of work or sports return, complications and patient satisfaction was observed. In our cohort, almost two thirds of (second stage ORIF) patients underwent SA. Therefore, PA may the superior choice for patients with complex midfoot injuries with massive articular damage or severe soft tissue injuries [45]. This may shorten the treatment course, reduce the risk of recurrent dislocation and progressive painful arthritis.

Despite strong effort, staged surgery aimed at limb salvage has shown to result in poor to fair outcome with a high risk of SA [25–27, 40]. Although amputation was averted in the vast majority, it comes at a cost of limited function, inability to return to previous daily activities including work and sports and chronic pain. An important option to consider at the start as well as during the course of treatment is whether limb reconstruction or salvage surgeries are (still) feasible. If not, objective reasoning may suggest that some patients are better treated with amputation [7]. However, early amputation is often perceived as catastrophic, whereas attempts at reconstruction may be more readily accepted. It is imperative to underscore the potential necessity for multiple surgeries required for reconstruction (even years after trauma), hospital admissions, long-term rehabilitation with poor functional outcome more often than not. Nevertheless, total costs of reconstruction (treatment and invalidity pensions) are lower compared to patients who undergo below knee amputation [46, 47]. Final functional outcome and patient satisfaction rates for both approaches are comparable and thus could be relevant in shared decision-making [7, 16, 46, 47].

### Limitations

This is the first study on long-term functional and validated patient-reported outcomes of patients after multi-stage treatment for complex Lisfranc and Chopart injuries. Previous literature was limited to a maximum of two surgeries and had a serious risk of bias with very low certainty of evidence. Data syntheses was not performed. There is potential bias due to the very low incidence of this injury, which led to a retrospective design, small sample size and the lack of comparable control group with the risk of selection bias. It was observed that most patients sustained combined Lisfranc and Chopart injuries, thereby constituting a larger group of severe midfoot trauma. Results may also have been skewed by the fact that the worst injuries were referred to our academic, foot and ankle trauma expertise center.

## Conclusion

Complex Lisfranc and Chopart injuries are very rare yet continue to be an important source of pain and disability. Due to the low incidence, high complexity, high risk of complications and poor to fair outcome of these complex midfoot injuries, early referral to dedicated foot surgeons is recommended. The aim is to achieve a functional, plantigrade foot that will allow the patient to resume pre-trauma activities, albeit with adaptations to foot wear, work and exercise. However, this study demonstrated that two thirds of second stage ORIF patients underwent SA. Primary arthrodesis may thus be the better choice as the second stage surgical plan.

## Electronic supplementary material

Below is the link to the electronic supplementary material.

Supplementary Material 1

Supplementary Material 2

## Data Availability

No datasets were generated or analysed during the current study.
