# Supplementary figures and images for: Long-term results of staged management of complex lisfranc and chopart injury: a retrospective cohort study and systematic literature review
Source: Eur J Trauma Emerg Surg. 2025 Jan 24;51(1):49. doi: 10.1007/s00068-024-02747-w (PMC11761783; doi:10.1007/s00068-024-02747-w)

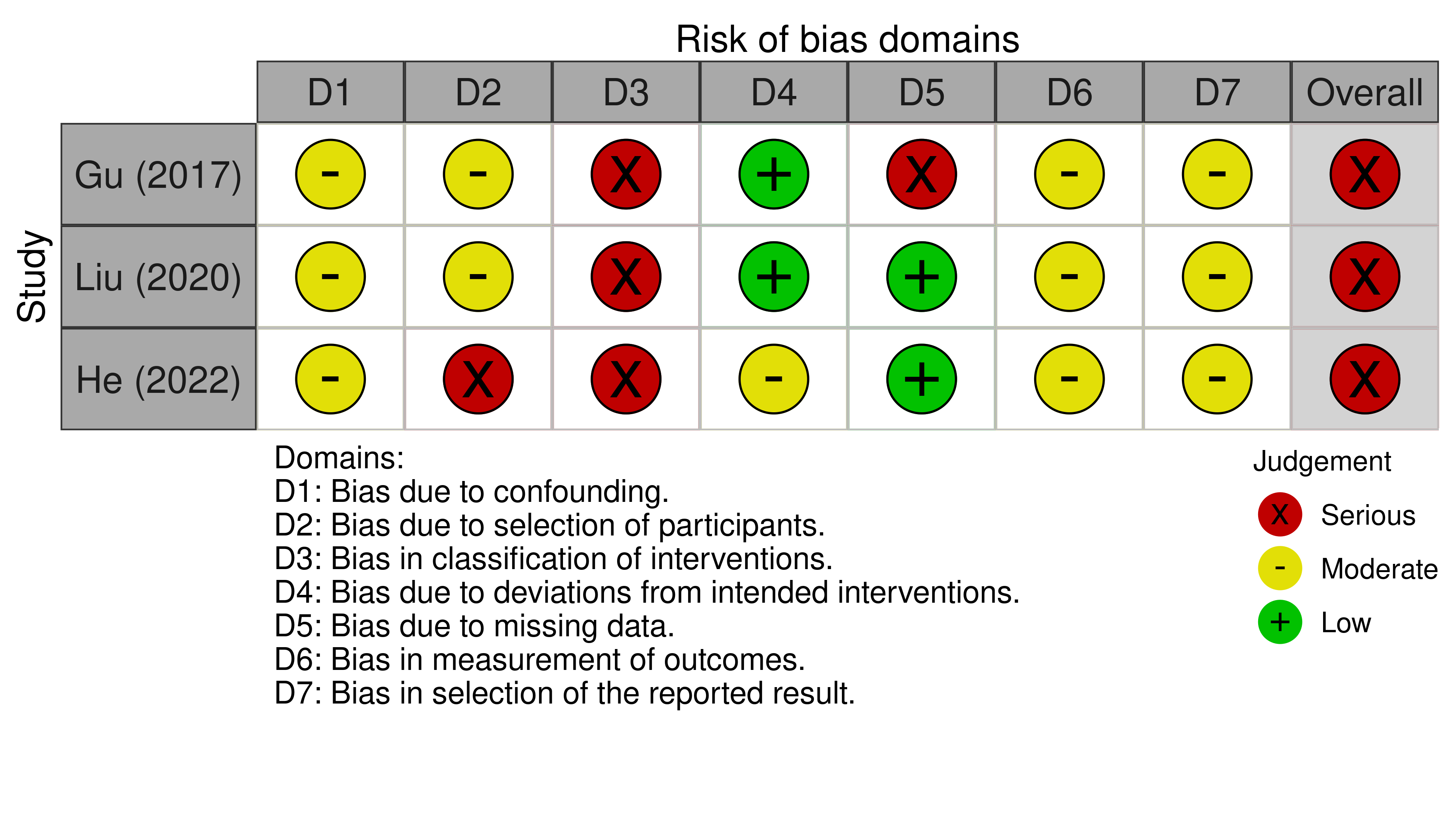

Supplement: Supplementary file 2 — Supplementary Material 2 [file 68_2024_2747_MOESM2_ESM.png]
